# Randomized clinical trial comparing the effects of an asynchronous mobile application to guided brief cognitive behavioral therapy for managing anxiety among medical students

**DOI:** 10.47626/2237-6089-2023-0713

**Published:** 2025-04-07

**Authors:** Andrian Fajar Kusumadewi, Carla Raymondalexas Marchira, Doni Widyandana, Ronny Tri Wirasto

**Affiliations:** 1 Universitas Gadjah Mada Dr Sardjito General Hospital Faculty of Medicine, Public Health and Nursing Yogyakarta Indonesia Department of Psychiatry, Faculty of Medicine, Public Health and Nursing, Dr Sardjito General Hospital, Universitas Gadjah Mada, Yogyakarta, Indonesia.; 2 Universitas Gadjah Mada Dr Sardjito General Hospital Faculty of Medicine, Public Health and Nursing Yogyakarta Indonesia Department of Medical Education and Bioethics, Faculty of Medicine, Public Health and Nursing, Dr Sardjito General Hospital, Universitas Gadjah Mada, Yogyakarta, Indonesia.

**Keywords:** Medical students, anxiety, mobile application, psychotherapy, brief CBT

## Abstract

**Objective:**

Medical students are a population at increased risk for anxiety due to their demanding schedule and concerns about potential stigmatization, which often discourage them from seeking help. It has been reported that the coronavirus disease (COVID-19) pandemic worsened this issue by restricting social interaction and mobility. An innovative method has been developed to address this problem, known as the asynchronous Digital Cognitive Education Gadjah Mada Anxiety Intervention for Medical Students (DCE GAMA-AIMS). Compared to traditional therapy, this modality can be accessed independently without the guidance of a therapist. The objective of this study was to compare the effectiveness of DCE GAMA-AIMS compared to therapist-guided brief cognitive behavioral therapy (bCBT) for reducing anxiety scores.

**Methods:**

A non-blinded randomized clinical trial (RCT) was conducted with 66 medical students. The participants were equally divided into two groups, an intervention and a control group. The intervention group was given DCE GAMA-AIMS, while guided bCBT was administered to the controls. The data obtained were analyzed using independent *t* tests and analysis of variance (ANOVA).

**Results:**

The application had a significant effect, reducing anxiety scores from the 2nd week (M Taylor Manifest Anxiety Scale [TMAS] = 18) to the 8th week (M TMAS = 13). A faster and more significant improvement was observed in the intervention group from the 1st to the 2nd week compared to the controls, who began to improve in the 4th week. Furthermore, the intervention group had larger effect size (1.32) compared to the control (0.79) from the 1st to 8th weeks.

**Conclusion:**

Asynchronous DCE GAMA-AIMS and guided bCBT both reduced TMAS scores in medical students with anxiety, but DCE GAMA-AIMS yielded a larger effect size.

## Introduction

Medical students are a population at elevated risk of mental health disorders, particularly anxiety, due to the extended duration of their education and demanding curricula.^1^ Previous reports have shown a 33.8% global prevalence of anxiety among this population. Furthermore, this percentage significantly exceeds prevalence in the general population and represents a marked increase compared to previous studies that have reported a rate of 11.5%.^2,3^ In the broader context of global demographics, the outbreak of the coronavirus disease (COVID-19) pandemic caused an increase in the number of individuals affected by anxiety. Incidence increased by up to 25%, with an overall prevalence of 47% during the first year of the pandemic. The most pronounced increase was observed among college students, with a rate of 81.8%.^4,5^ According to a previous study, failure to treat anxiety can be detrimental and disrupt productivity, leading to reductions in quality of life.^6^

Various modalities for treating anxiety are presently available, comprising pharmacotherapy, neurofeedback, psychoeducation, cognitive therapy, behavioral therapy, mindfulness, relaxation methods, and religious therapy, but several limitations still persist within these treatment options. These limitations include the potential side effects of medication, constraints on access to healthcare facilities, strict schedules, stigma, and uneven distribution of therapists. Furthermore, the condition can reduce medical students’ motivation to seek treatment as patients, limit reach of therapy services, lower patient compliance, and increase dropout rates.^7,8^

Brief cognitive behavioral therapy (bCBT) has shown effectiveness as an alternative treatment for social anxiety disorder (SAD) among medical students. This option has also shown efficiency in situations where a shortage of qualified therapists exists, but it still has several drawbacks.^9^ To address the challenges posed by globalization and constraints on existing therapy, there is a need for therapy modalities that are cost-effective, easily accessible, efficacious, free from stigma, and consistent with technological advancements. Several related studies have recommended strategies, such as comparing smartphone-accessible interventions to existing treatments, investing in user-centered design reports, and exploring the applicability and efficacy of other theories/models.^10,11^ Therefore, this study aims to assess the effectiveness of mobile application-based psychotherapy compared to conventional face-to-face psychotherapy (guided synchronous bCBT) for mitigating anxiety among medical students in Indonesia.

## Methods

### Participants

This study was a single-center, non-blinded, randomized clinical trial (RCT), with a purposive sampling method. The participants of the randomized controlled phase were recruited from the Faculty of Medicine, Public Health, and Nursing at Gadjah Mada University Yogyakarta, Indonesia, from April to June 2022. This pilot study was conducted in line with the Health Promoting University (HPU) program initiated by Gadjah Mada University for medical students, who were known to be at high risk of experiencing anxiety.^1-3^

The sample population consisted of all undergraduate or professional education students (batch 2017-2021) who experienced anxiety symptoms based on the General Anxiety Disorder-7 (GAD-7) questionnaire and indicated their willingness to participate in the procedures by voluntarily providing informed consent. The diagnosis and psychiatric condition of having anxiety were established by psychiatric trainees who were supervised by psychiatrists. Furthermore, this process was conducted using a symptomatic method to diagnose anxiety based on Diagnostic and Statistical Manual of Mental Disorders, 5th edition (DSM-5) to exclude subjects who met the exclusion criteria. Participants receiving therapy for psychiatric conditions, having a history of drug abuse, or showing other symptoms leading to a more severe disorder (subjects with psychotic symptoms, severe mood disorder, and psychiatric emergencies such as self-harm and suicidal thoughts) were excluded. A minimum sample size of 36 in each group was required to detect a large effect size with a power of 90% and an alpha error of 0.05.^12^

Among the population of 994 students, 568 were willing to complete the GAD-7 questionnaire and 179 met the GAD-7 criteria (GAD score ≥ 5). Furthermore, only 86 medical students agreed to participate in the study and were divided into two groups. In the control group, 43 participants were given 8 weeks of brief bCBT guided by a therapist during Zoom calls. The intervention group was given Asynchronous Digital Cognitive Education (DCE), delivered via a mobile-based online application. A total of 66 students, 33 in each group, were able to complete the study, while 20 dropped out during the treatment. During the procedures, 10 participants in the intervention group and nine participants in the control were lost to follow-up because they could not be contacted through chat or telephone calls. Meanwhile, one respondent in the control group, as monitored and evaluated by the psychiatric trainee, showed worsening symptoms (psychotic) and was referred to a psychiatrist in the hospital for further treatment and assessment.

### Measures

The tool used for anxiety screening was the GAD-7, with cutoff points of 5, 10, and 15 indicating mild, moderate, and severe levels, respectively.^13^ The participants included in this study were medical students from batch 2017 to 2021 who had GAD scores ≥ 5 and were later diagnosed with anxiety based on DSM-5 criteria.^14^ Anxiety scores were assessed with the Taylor Manifest Anxiety Scale (TMAS) at baseline before the intervention and evaluated each week for a total of 8 weeks. The TMAS is a self-report questionnaire comprising 50 items and increasing TMAS scores correlated with higher levels of anxiety.^15^ All data collection and monitoring were carried out using online questionnaire forms and meetings.

### Intervention

#### Guided bCBT

**Figure 1:**
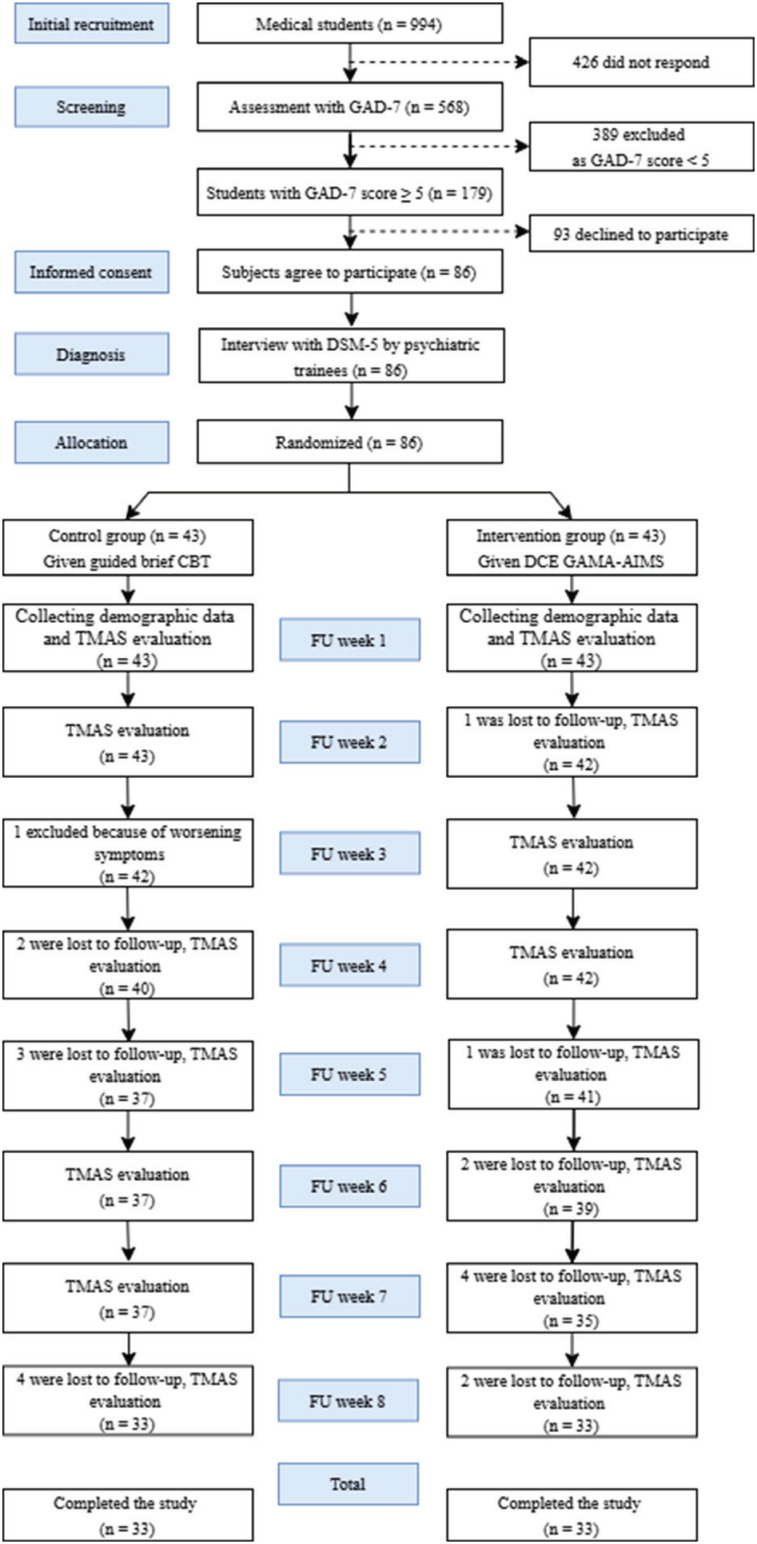
Flow of participants through sample allocation. CBT = cognitive behavioral therapy; DCE GAMA-AIMS: Digital Cognitive Education Gadjah Mada Anxiety Intervention for Medical Students; DSM-5 = Diagnostic and Statistical Manual of Mental Disorders, 5th edition; FU = follow up; GAD-7 = General Anxiety Disorder-7; TMAS = Taylor Manifest Anxiety Scale.

Guided bCBT was given to the control group as gold standard non-pharmacological treatment. Brief psychotherapy consisted of eight weekly bCBT sessions, each comprising a 1-hour online meeting, guided by psychiatry trainees who already had the clinical authority to manage patients with anxiety. The psychiatry trainees were trained by a professional clinical psychologist with more than 5 years of experience in performing CBT and directly supervised by psychiatrists involved in this study. The comparison to bCBT treatment aimed to strengthen the conclusion that therapy obtained by the intervention group produced significant effects and output. Furthermore, it could minimize errors, such as the placebo effect and threats of validity in making conclusions about intervention results (Figure 1).

#### Asynchronous DCE Gadjah Mada Anxiety Intervention for Medical Students (GAMA-AIMS)

**Figure 2:**
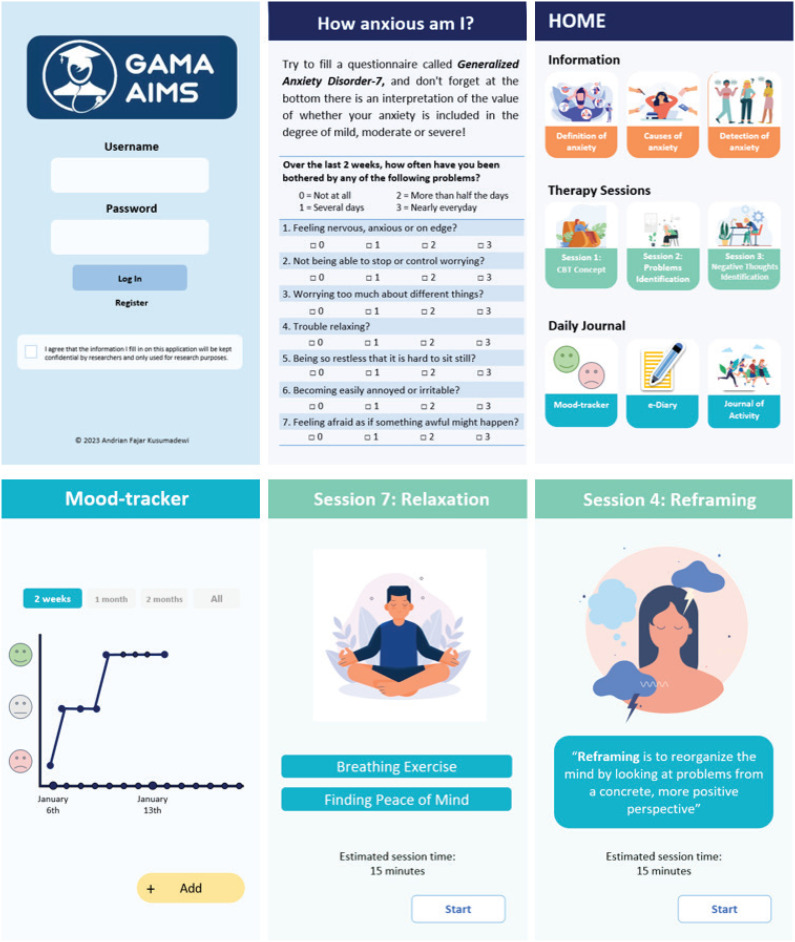
Interface of the GAMA-AIMS Application. This image shows the login page, list of menus, and types of menus in the application

Asynchronous DCE-GAMA AIMS was the intervention modality designed for this study. Furthermore, it was created based on cognitive psychoeducational methods and delivered digitally through the GAMA-AIMS smartphone-based application. During the process of psychotherapy, patients actively engaged with the material through a device used independently without the presence of a therapist (unguided real-time self-help). The application was created in collaboration with medical education experts, psychiatrists, clinical psychologists, and mobile application developers. The application menu was divided into three parts, comprising information, therapy, and daily journal sections. The information section contained an explanation of anxiety covering definition, signs, and symptoms, as well as how to seek help. The therapy section was developed based on Beckian Cognitive Therapy and consisted of eight submenus, namely, explanation of the therapy concept, problem identification, negative thoughts identification, reframing, behavioral activation, problem-solving, relaxation, and evaluation. The daily journal section consisted of a mood tracker, e-diary, and journal of activity (Figure 2). This module has passed various trials to test its internal validity, reliability, safety, and usability.^16,17^

### Statistical analysis

The sample characteristics variables were tested using independent *t* tests. The extent of symptom change over an 8-week follow-up period within the two groups was determined using linear mixed model analysis of variance (ANOVA). Subsequently, data were analyzed using SPSS version 25 (IBM Corp, Armonk, NY, USA).

### Ethical considerations

This study was approved by the Medical and Health Research Ethics Commission at the Faculty of Medicine, Public Health and Nursing, Gadjah Mada University (KE/FK/1051/EC/2021, of September 21, 2021). An amendment to the ethics statement was made on January 9, 2023 (KE/FK/0048/EC), and the last ethical amendment was made on June 5, 2023 (KE/FK/0048/EC).

The study participants provided online consent and the procedures were carried out based on the ethical principles of human studies, as outlined in the Declaration of Helsinki.

## Results

### Baseline characteristics

The average age of the respondents was 20.45 years (standard deviation [SD] = 0.71), and 78.79% of them were female. The results showed that there were no statistically significant differences between intervention and control groups in terms of anxiety severity or the two baseline characteristics (Table 1).

**Table 1:** Demographic data

Variables		Guided bCBT	Asynchronous DCE	Z/X²	p-value
Age, mean (SD)		20.58 (1.48)	20.33 (1.22)	-0.430	0.667
Sex female, n (%)		26 (78.79)	26 (78.79)	0.00	1.00
				
GAD-7 category, n (%)				1.50	0.471
	Mild	15 (45.5)	11 (33.3)		
	Moderate	11 (33.3)	11 (33.3)		
	Severe	7 (21.2)	11 (33.3)		

bCBT = brief cognitive behavioral therapy; DCE = Digital Cognitive Education; GAD-7 = General Anxiety Disorder-7; SD = standard deviation.

### Treatment outcome and effect size

Linear mixed model ANOVA was used to assess the extent of symptom change over an 8-week follow-up period within each of the two groups. Considering that Mauchley's test of sphericity was significant, the Greenhouse-Geisser correction was applied. Furthermore, an important treatment interaction between group and time was observed in the self-report outcome (TMAS), yielding a statistically significant result (F [4.39, 68.10] = 3.65, p = 0.005).

Within-group analyses were conducted using paired sample *t* tests (Table 2), showing a significant improvement in the DCE group from the 1st week to the 2nd week. The results showed that this improvement in the TMAS measure continued up to the 8th week (t_[33]_ = 2.66-6.71, p < 0.05). In contrast, the guided bCBT group did not show significant recovery during the 2nd (t_[33]_ = 1.94, p = 0.062) or the 3rd week (t_[33]_ = 1.63, p = 0.113). However, subsequent paired sample *t* tests for the guided bCBT group did show significant improvement from the 4th week to the 8th week, in comparison to the initial TMAS score (t_[33]_ = 2.68-5.90, p < 0.05).

**Table 2:** Treatment outcomes

	TMAS 1 M (SD)	TMAS 2 M (SD)	TMAS 3 M (SD)	TMAS 4 M (SD)	TMAS 5 M (SD)	TMAS 6 M (SD)	TMAS 7 M (SD)	TMAS 8 M (SD)
Guided bCBT	22.28 (5.80)	21.22 (5.45)	21.38 (5.77)	20.78 (5.91)	19.09 (5.98)	19.31 (5.84)	19.09 (6.41)	17.41 (6.38)
Asynchronous DCE	21.16 (6.50)	18.78 (6.68)	17.13 (6.25)	16.78 (6.49)	14.59 (7.36)	14.00 (7.11)	12.97 (6.82)	12.16 (7.06)
Between-group t value (p-value) df = 66	t = −0.731 (0.468)	t = −1.60 (0.115)	t = −2.82 (0.006)	t = −2.58 (0.012)	t = −2.68 (0.009)	t = −3.27 (0.002)	t = −3.70 (0.000)	t = −3.12 (0.003)
								
Within group t value (p-value) compared to TMAS 1								
Guided bCBT, df = 33		t = 1.94 (0.062)	t = 1.63 (0.113)	t = 2.68 (0.012)	t = 3.37 (0.002)	t = 5.46 (0.000)	t = 4.61 (0.000)	t = 5.90 (0.000)
Asynchronous DCE, df = 33		t = 2.66 (0.012)	t = 4.18 (0.000)	t = 3.86 (0.001)	t = 5.63 (0.000)	t = 6.17 (0.000)	t = 6.71 (0.000)	t = 6.39 (0.000)

bCBT = brief cognitive behavioral therapy; DCE = Digital Cognitive Education; df = degrees of freedom; SD = standard deviation; TMAS = Taylor Manifest Anxiety Scale.

Although the results indicated that Asynchronous DCE was superior to guided bCBT, additional analyses were carried out to determine the magnitude of the treatment effect within each group. This was achieved by calculating within-group effect sizes using Cohen's *d* formula, as presented in Table 3. Following Cohen's established classification of effect sizes into small (0.20-0.49), medium (0.50-0.79), and large (0.80 and above), the large and medium categories were observed in this study for Asynchronous DCE (1.32), and guided bCBT (0.79) from the 1st to the 8th week, respectively.

**Table 3:** Effect sizes (Cohen's *d*)

Assessment	Guided bCBT	Asynchronous DCE
TMAS		
1st-8th week	0.79	1.32
Between-group effect size (8th week)	0.78	

bCBT = brief cognitive behavioral therapy; DCE = Digital Cognitive Education; TMAS = Taylor Manifest Anxiety Scale.

## Discussion

This study provides evidence supporting the effectiveness of Asynchronous DCE as a treatment for anxiety among medical students in Yogyakarta, Indonesia. Participants who received DCE showed significant improvements in symptoms, as indicated by the TMAS score. Furthermore, the treatment effects of the DCE intervention were found to be superior to those of guided bCBT. The analysis showed a large effect size for the 8-week Asynchronous DCE treatment, while a moderate effect size was observed for guided bCBT.

These results were consistent with previous studies using an Asynchronous psychotherapy protocol, but those reports incorporated additional psychological measures and used a general health information module as a control. For example, Batterham et al.^18^ observed a significant impact of the self-guided intervention, reducing anxiety after 4 weeks of treatment.

In another study, a treatment intervention was implemented consisting of nine 10-week modules comprising psychoeducation, internet CBT (iCBT), and physical activity promotion, whereas the intervention in the present study consisted of 8 weekly sessions. The earlier study showed a significant secondary effect on anxiety sensitivity (measured by the Anxiety Sensitivity Index [ASI]), worry, and depression. However, Christensen^19^ stated that the primary outcome measure for anxiety, assessed using the GAD-7, did not show superiority over the placebo website condition. Although it is challenging to pinpoint the exact cause of this difference, several factors could contribute. Several studies have stated that while the GAD-7 was effective in detecting various anxiety disorders, it could possibly be a less sensitive measure of changes compared to the ASI.

The reduction in symptoms was consistent with Miller et al.,^20^ in that the anxiety levels of participants, assessed using the GAD-7, decreased among the majority of participants (out of a total of 21 respondents) who engaged in digital CBT weekly from baseline to the end of the 6th week of intervention. Levels also continued to decrease during the follow-up period, up to the 10th week. According to Kackzurkin et al.,^21^ exposure therapy combined with relaxation methods and CBT yielded better outcomes among affected individuals.

The results of this study were not consistent with Carl et al.,^22^ McCloud et al.,^16^ and Ponzo et al.^23^ Carl et al.^22^ stated that there was a significant reduction in anxiety levels after use of digital CBT at the 6th week after randomization. Meanwhile, McCloud et al.,^16^ using the FeelStressFree application, and Ponzo et al.,^23^ using RCT based on the BioBase application, found a decrease at the 4th week.^1^ Based on these results, Asynchronous DCE's ability to reduce the condition at the 7th week remained superior compared to conventional CBT, which required a minimum of 10 weeks for symptom improvement.^24,25^ Dafroyati^25^ also identified a decrease in TMAS anxiety levels, with the majority experiencing mild anxiety after 10 sessions (10 hours) of conventional CBT. Several reports considered psychoeducation therapy effective because it was in line with the medical model of illness, emphasizing that mental conditions could be managed and treated in a similar manner as physical conditions.^26^

During the 8-week intervention based on relaxation methods and cognitive therapy, a reduction in anxiety levels was successfully achieved. However, the results showed that the conditions experienced by the participants did not completely disappear. This result is consistent with a previous report of an internet intervention on a campus, where the average level of stress did not decrease after intervention. This was due to a lack of time to implement the methods, as well as the presence of workload and chronic stress that continued to provoke symptoms.^27^ Based on the weekly intervention results and the results after use of the application over 8 weeks, use of the GAMA-AIMS Asynchronous DCE intervention at least once a week could reduce the severity of participants’ conditions starting from the 5th week. Furthermore, a significant decrease was observed when the application was used for 7 weeks.

It was important to emphasize that the interventions used in this study were conducted weekly over a total of 8 weeks based on psychoeducation principles. Furthermore, psychoeducation guidelines recommended a minimum of one intervention per week, lasting for 6-12 weeks to achieve results similar to psychotherapy.^28^ The difference in results can be attributed to the format of the GAMA-AIMS application menu, which was specifically tailored to health science students and the language and content were more understandable for the participants.

The results obtained were similar to the intervention results of the Healthy Mind application. In the first 2 weeks, users accessed the application an average of 2 times, with an average duration of 19 minutes per access, showing a wide variation in login frequency and duration (users could access the application up to 26 times).^29^ The choice to provide access to the anxiety psychoeducation module through an application was based on previous studies and applications that had shown better frequency of usage compared to web-based intervention. Compared to the web-based module Healthy Paths, the Healthy Mind application was accessed twice as often, with slightly shorter durations per login.^29^ Previous studies also indicated that people tended to use mobile applications for very short periods, considering their habit of using smartphones during leisure time. The application must be quickly accessible, have simple interactions, and support one or a limited set of tasks, preferably related to previous conditions and intervention.^30^

Asynchronous DCE GAMA-AIMS therapy presented a multitude of advantages in comparison to alternative therapy methods. This therapy was able to reduce anxiety scores by enabling users to frequently engage in therapy sessions on a mobile application weekly. Given its digital nature, this mobile application offered easier access and demonstrated the potential to be the primary resource for medical students experiencing anxiety. Furthermore, it was in line with the demands of medical students who are subjected to heavy academic loads and tight schedules and facilitated ease of access compared to conventional or online CBT. Conventional or online CBT requires appointments with therapists, which necessitates coordination of schedules either through use of the Zoom application or in face-to-face meetings. Considering the prevailing stigma surrounding mental health disorders, the mobile application provides medical access to mental health services, thereby facilitating treatment of anxiety. This indicates that it could be part of the medical faculty's mental health provision for their students.^31,32^

In this study, a dropout rate of 23.25% was observed over the course of the treatment. Although there is no absolute consensus or recommendation, several journals mentioned that a dropout rate exceeding 20% potentially affected the quality of results in RCTs. This was observed particularly in the aspects of statistical power, bias, and generalizability, especially when the distribution of missing data varied significantly between the two groups.^33-35^ Other references stated that it was very difficult to achieve a < 20% dropout rate, especially in non-pharmacological intervention (e.g., psychotherapy, including CBT) with repeated outcome measurements and long-term therapy duration (> 4 weeks), as implemented in this study. Therefore, a rate below 30% could still be considered acceptable.^33,34,36^

A meta-analysis examining attrition in CBT intervention studies reported an average weighted dropout rate during treatment of 26.2%, which is slightly higher compared to this study. CBT in the e-therapy format exhibited a higher average, reaching 34.2%.^37^ The results identified several factors that could lead to discontinuation of participation in the intervention, including time-related factors (especially for the control group), personal interest, and commitment to therapy, as well as perceived improvements in symptoms.^34,35^ However, due to the high rate of informal dropout (loss to follow-up without formal notice), the exact cause of the action could not be further explored.

Although the DCE GAMA-AIMS Asynchronous Psychotherapy had a significant effect on reducing anxiety scores among medical students, some limitations must be acknowledged, such as a relatively small sample size drawn from a single center. This indicates that further studies should use larger populations in various settings to obtain more generalizable results. In this study, there was no physical examination or biological markers related to anxiety. Data collection mostly relied on self-report assessments and online meetings due to pandemic conditions. This led to sub-optimal monitoring and evaluation, as well as loss of participants to follow-up. Therefore, future studies are advisable to determine the long-term effect of therapy and its role in preventing relapse.

## Conclusion

In conclusion, Asynchronous DCE GAMA-AIMS and online guided bCBT could both reduce TMAS scores in medical students with anxiety. However, it was observed that DCE GAMA-AIMS yielded a slightly greater effect size. This indicates that the application could be considered as an accessible alternative initial therapy or self-help. Further studies and developments are necessary to maximize effect and generalizability before implementing interventions.

## References

[1] Jafri SAM, Zaidi E, Aamir IS, Aziz HW, Imad-ud-Din, Shah MAH. (2017). Stress level comparison of medical and non-medical students: a cross-sectional study done at various professional colleges in Karachi, Pakistan. Acta Psychopathol.

[2] Tian-Ci Quek T, Wai-San Tam W, X. Tran B, Zhang M, Zhang Z, Su-Hui Ho C, Chun-Man Ho R (2019). The global prevalence of anxiety among medical students: a meta-analysis. Int J Environ Res Public Health.

[3] Chandavarkar U, Azzam A, Mathews CA (2007). Anxiety symptoms and perceived performance in medical students. Depress Anxiety.

[4] World Health Organization (2022). World mental health report: transforming mental health for all.

[5] Hosen I, Al-Mamun F, Mamun MA (2021). Prevalence and risk factors of the symptoms of depression, anxiety, and stress during the COVID-19 pandemic in Bangladesh: A systematic review and meta-analysis. Glob Ment Health.

[6] Hendriks SM, Spijker J, Licht CMM, Hardeveld F, de Graaf R, Batelaan NM (2016). Long-term disability in Anxiety Disorders. BMC Psychiatry.

[7] Kambeitz-Ilankovic L, Rzayeva U, Völkel L, Wenzel J, Weiske J, Jessen F (2022). A systematic review of digital and face-to-face cognitive behavioral therapy for depression. npj Digit Med.

[8] Hofmann SG, Ellard KK, Siegle GJ (2012). Neurobiological correlates of cognitions in fear and anxiety: A cognitive-neurobiological information-processing model. Cognit Emot.

[9] Samantaray NN, Nath B, Behera N, Mishra A, Singh P, Sudhir P (2021). Brief cognitive behavior group therapy for social anxiety among medical students: A randomized placebo-controlled trial. Asian J Psychiatry.

[10] Oliveira C, Pereira A, Vagos P, Nóbrega C, Gonçalves J, Afonso B (2021). Effectiveness of mobile app-based psychological interventions for college students: a systematic review of the literature. Front Psychol.

[11] Newman MG, Jacobson NC, Rackoff GN, Bell MJ, Taylor CB (2020). A randomized controlled trial of a smartphone-based application for the treatment of anxiety. Psychother Res.

[12] Howell AN, Rheingold AA, Uhde TW, Guille C (2019). Web-based CBT for the prevention of anxiety symptoms among medical and health science graduate students. Cogn Behav Ther.

[13] Locke AB, Kirst N, Shultz CG (2015). Diagnosis and management of generalized anxiety disorder and panic disorder in adults. Am Fam Physician.

[14] American Psychiatric Association (2013). Diagnostic and Statistical Manual of Mental Disorders.

[15] Mancevska S, Bozinovska L, Tecce J, Pluncevik-Gligoroska J, Sivevska-Smilevska E (2008). Depression, anxiety and substance use in medical students in the Republic of Macedonia. Bratisl Lek Listy.

[16] McCloud T, Jones R, Lewis G, Bell V, Tsakanikos E (2020). Effectiveness of a mobile app intervention for anxiety and depression symptoms in university students: randomized controlled trial. JMIR Mhealth Uhealth.

[17] Lee RA, Jung ME (2018). Evaluation of an mHealth App (DeStressify) on University Students’ Mental Health: Pilot Trial. JMIR Mental Health.

[18] Batterham PJ, Calear AL, Farrer L, Gulliver A, Kurz E (2021). Efficacy of a Transdiagnostic Self-Help Internet Intervention for Reducing Depression, Anxiety, and Suicidal Ideation in Adults: Randomized Controlled Trial. J Med Internet Res.

[19] Christensen H, Batterham P, Calear A (2014). Online interventions for anxiety disorders. Curr Opin Psychiatry.

[20] Miller CB, Gu J, Henry AL, Davis ML, Espie CA, Stott R (2021). Feasibility and efficacy of a digital CBT intervention for symptoms of Generalized Anxiety Disorder: A randomized multiple-baseline study. J Behav Ther Exp Psychiatry.

[21] Kaczkurkin AN, Foa EB (2015). Cognitive-behavioral therapy for anxiety disorders: an update on the empirical evidence. Dialogues Clin Neurosci.

[22] Carl JR, Miller CB, Henry AL, Davis ML, Stott R, Smits JAJ (2020). Efficacy of digital cognitive behavioral therapy for moderate-to-severe symptoms of generalized anxiety disorder: A randomized controlled trial. Depress Anxiety.

[23] Ponzo S, Morelli D, Kawadler JM, Hemmings NR, Bird G, Plans D (2020). Efficacy of the Digital Therapeutic Mobile App BioBase to Reduce Stress and Improve Mental Well-Being Among University Students: Randomized Controlled Trial. JMIR Mhealth Uhealth.

[24] Aslan Y (2020). The effectiveness of cognitive behavioral therapy (CBT) on generalized anxiety disorder (GAD). ResearchGate.

[25] Dafroyati Y, Widyastuti R, Suparji S (2022). Cognitive behavior therapy lowers anxiety levels of pregnant women during the COVID-19 pandemic at Sikumana Health Center. Open Access Maced J Med Sci.

[26] Colom F (2011). Keeping therapies simple: psychoeducation in the prevention of relapse in affective disorders. Br J Psychiatry.

[27] Fleischmann RJ, Harrer M, Zarski AC, Baumeister H, Lehr D, Ebert DD (2018). Patients’ experiences in a guided Internet- and App-based stress intervention for college students: A qualitative study. Internet Interventions.

[28] Sarkhel S, Singh OP, Arora M (2020). Clinical Practice Guidelines for Psychoeducation in Psychiatric Disorders General Principles of Psychoeducation. Indian J Psychiatry.

[29] Morrison LG, Geraghty AWA, Lloyd S, Goodman N, Michaelides DT, Hargood C (2018). Comparing usage of a web and app stress management intervention: An observational study. Internet Interventions.

[30] Vaish R, Wyngarden K, Chen J, Cheung B, Bernstein MS (2014). Proceedings of the SIGCHI Conference on Human Factors in Computing Systems.

[31] Venkatesan A, Rahimi L, Kaur M, Mosunic C (2020). Digital Cognitive Behavior Therapy Intervention for Depression and Anxiety: Retrospective Study. JMIR Ment Health.

[32] Cohen D (2015). Supporting medical students with mental health conditions.

[33] Fewtrell MS, Kennedy K, Singhal A, Martin RM, Ness A, Hadders-Algra M (2008). How much loss to follow-up is acceptable in long-term randomised trials and prospective studies?. Arch Dis Child.

[34] Dixon LJ, Linardon J (2020). A systematic review and meta-analysis of dropout rates from dialectical behaviour therapy in randomized controlled trials. Cogn Behav Ther.

[35] Christensen H, Griffiths KM, Farrer L (2009). Adherence in Internet Interventions for Anxiety and Depression. J Med Internet Res.

[36] Furlan AD, Pennick V, Bombardier C, Van Tulder M. (2009). 2009 Updated Method Guidelines for Systematic Reviews in the Cochrane Back Review Group: Spine.

[37] Fernandez E, Salem D, Swift JK, Ramtahal N (2015). Meta-analysis of dropout from cognitive behavioral therapy: Magnitude, timing, and moderators. J Consult Clin Psychol.

